# Bacterial Enzymes and Antibiotic Resistance

**Published:** 2018

**Authors:** A. M. Egorov, M. M. Ulyashova, M. Yu. Rubtsova

**Affiliations:** Chemistry Faculty, M.V. Lomonosov Moscow State University, Leninskie gori, 1, bldg. 3, Moscow, 119991, Russia

**Keywords:** antibiotic resistance, enzymes, mutant forms, antibiotics

## Abstract

The resistance of microorganisms to antibiotics has been developing for more than 2 billion years and is widely distributed among various representatives of the microbiological world. Bacterial enzymes play a key role in the emergence of resistance. Classification of these enzymes is based on their participation in various biochemical mechanisms: modification of the enzymes that act as antibiotic targets, enzymatic modification of intracellular targets, enzymatic transformation of antibiotics, and the implementation of cellular metabolism reactions. The main mechanisms of resistance development are associated with the evolution of superfamilies of bacterial enzymes due to the variability of the genes encoding them. The collection of all antibiotic resistance genes is known as the resistome. Tens of thousands of enzymes and their mutants that implement various mechanisms of resistance form a new community that is called “the enzystome.” Analysis of the structure and functional characteristics of enzymes, which are the targets for different classes of antibiotics, will allow us to develop new strategies for overcoming the resistance.

## INTRODUCTION

**Fig. 1:**
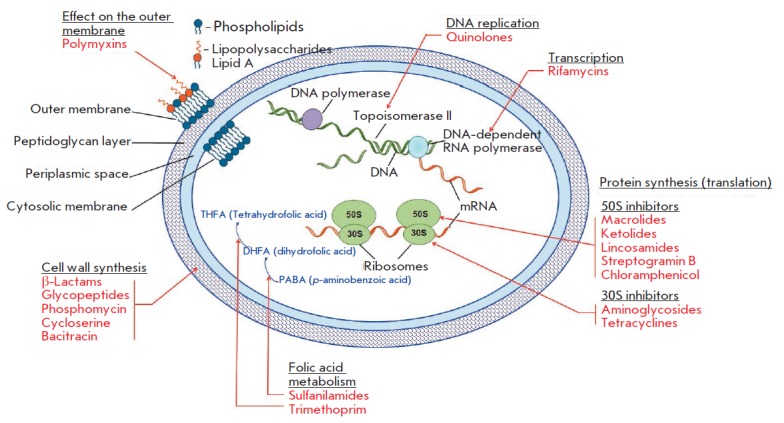
The main classes of antimicrobial drugs, their targets, and their effect on the main processes of vital activity of a bacterial cell

Antibiotic resistance of the causative agents of infectious diseases is a global problem in biology and medicine [1, 2]. Modern antimicrobial drugs (AMDs) represent the largest group of pharmaceutical drugs, including 16 classes of natural and synthetic compounds (*Fig. 1*).

Synthesis of antibiotics has existed in nature for more than 2 billion years. During all this time, bacteria have been developing mechanisms of resistance to their toxic action. Resistance may occur as an adaptive process unrelated to the structure of an antibiotic or develop as a result of the selection of resistant strains of microorganisms under the influence of antibiotics. The anthropogenic factors associated with the application of antibiotics in medicine and, especially, in agriculture since the mid-20^th^ century have led to a significant evolution of resistance mechanisms; the time it takes to develop resistance to new drugs has significantly reduced [3, 4].

**Fig. 2:**
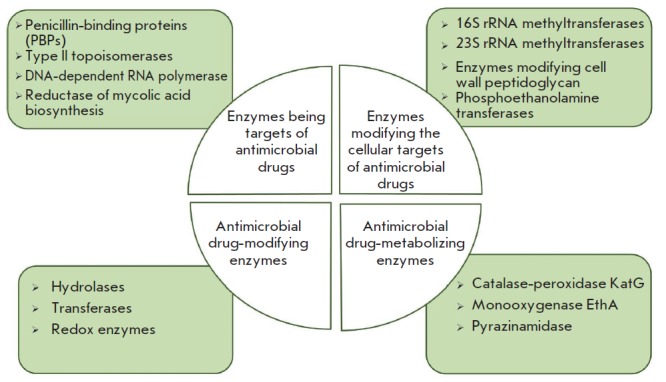
Classes of enzymes involved in various mechanisms of resistance to antimicrobial drugs

The role of bacterial enzymes in resistance development is rather versatile and involves several key mechanisms (*Fig. 2*) [5]. The enzymes involved in cell wall biosynthesis, as well as the synthesis of nucleic acids and metabolites, serve as a direct target for antibiotics. The resistance mechanism is associated with structural changes in these enzymes. Another mechanism is associated with the enzymatic modification of the structural elements affected by antibiotics: for example, modification of ribosomes by methyltransferases. A large group of enzymes modify or destroy the structure of antibiotics by inactivating them. Enzymes catalyzing metabolic processes and modifying AMDs in the form of prodrugs are also involved in resistance development.

The bacterial enzymes that determine resistance usually belong to large superfamilies; many of them originated from enzymes that originally had other functions [6]. The genes responsible for the synthesis of these enzymes and their mutational variability are often localized on mobile genetic elements, thus ensuring the rapid spread of resistance between microorganisms.

This review presents data on the functional features of the main classes and groups of the bacterial enzymes involved in the implementation of the mechanisms of bacterial resistance to AMDs.

**Fig. 3:**
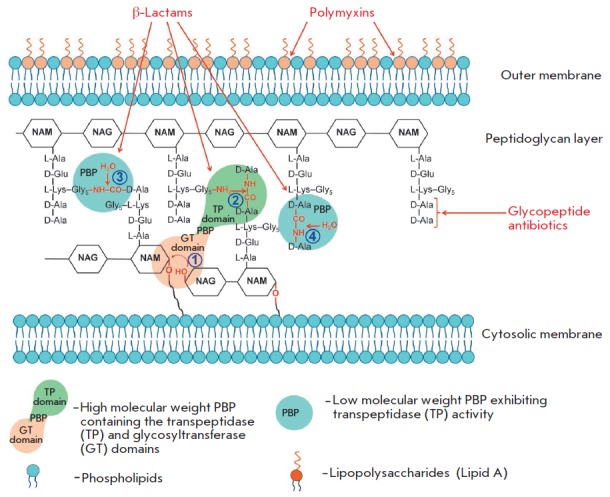
The structure of bacterial cell wall peptidoglycan and involvement of penicillin-binding proteins in different reactions of its synthesis: 1 – transglycosylation reaction, 2 – transpeptidation reaction, 3 – endopeptidation reaction, and 4 – carboxypeptidation reaction

## BACTERIAL ENZYMES AS THE TARGETS OF AMDs

**Penicillin-binding proteins**

Penicillin-binding proteins (PBPs) play a key role in the synthesis of peptidoglycan, the main component of bacterial cell walls. PBPs are the targets of β-lactam antibiotics. Peptidoglycan is a polymer consisting of alternating N-acetylglucosamine (NAG) and N-acetylmuramic acid (NAM) residues (*Fig. 3*). Peptides containing *L*-Ala, *D*-Glu, meso-diaminopimelic acid or *L-*Lys, and two *D*-Ala residues are attached to all NAM residues [7]. PBPs are bound to the inner cell membrane or found in free form in the cytosol [8, 9]. PBPs are divided into high-molecular-weight (> 50 kDa) proteins consisting of two domains and low-molecular-weight proteins ( < 50 kDa).

The N-terminal domain of high-molecular-weight PBP catalyzes transglycosylation reactions (sequential elongation of glycan chains by the addition of NAG-NAM-pentapeptide to the glycan backbone, 1 in *Fig. 3*). The C-terminal domain catalyzes transpeptidase reactions (cross-linking of peptide residues in two glycan chains, 2 in *Fig. 3*). Low-molecular-weight PBPs prevent cross-linking in peptidoglycan; they catalyze endopeptidase (hydrolysis of the peptide bond connecting two glycan chains, 3 in *Fig. 3*) and carboxypeptidase (hydrolysis of the bond in *D*-Ala-*D*-Ala dipeptide, 4 in *Fig. 3*) reactions.

The C-terminal domains of all PBPs are the targets of β-lactam antibiotics, which constitute more than half of all currently used AMDs [10]. These antibiotics contain a β-lactam ring, a structural analogue of *D*-Ala-*D*-Ala dipeptide, and, therefore, act as competitive inhibitors of PBPs. The interaction between the carbonyl group in the β-lactam ring and the hydroxyl group of serine in the active center of a PBP gives rise to an inactive acylated form of the enzyme. Irreversible inhibition disrupts the synthesis of the bacterial cell wall [9, 10].

**Fig. 4:**
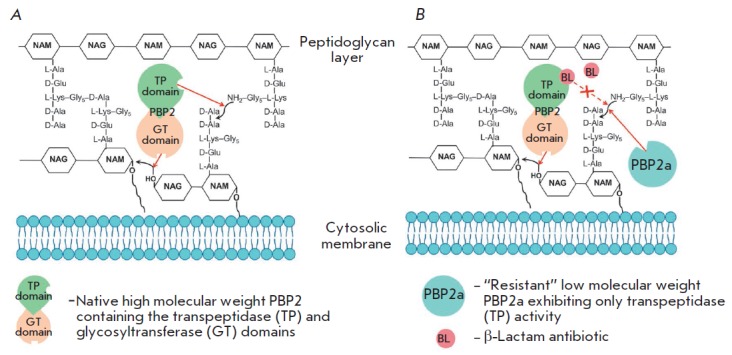
The role of penicillin-binding proteins in the resistance of Gram-positive bacteria to β-lactam antibiotics. A – sensitive strain, B – resistant strain

The main reasons why Gram-positive bacteria develop resistance to β-lactam antibiotics include mutations in native PBPs, their hyperproduction, and the synthesis of new PBPs that are insensitive to inhibition by β-lactams [11]. Today, the spread of *Staphylococcus aureus *strains resistant to methicillin and other semisynthetic penicillins and cephalosporins poses a threat [12]. Resistance is determined by expression of the fifth enzyme, PBP2a (in addition to the four native PBPs), which has low affinity for β-lactam antibiotics and exhibits transpeptidase activity only. *Figure 4* shows the resistance mechanism: without an antibiotic, both domains of a high-molecular-weight PBP are involved in peptidoglycan biosynthesis (*A*); only the glycosyltransferase domain remains active in a high-molecular-weight PBP in the presence of an antibiotic, while the transpeptidase domain is acylated and does not form crosslinks. It is the acquired low-molecular-weight PBP2a (B) that exhibits transpeptidase activity in the resistant strain. As a result, cell viability is restored.

PBP2a enzymes are encoded by the genes mecA [13] or mecC [14]. The *mecA *and *mecC *genes, together with the genes regulating their expression (*mecI, mecR1 *and *mecR2*), are the components of the mobile genetic element of the staphylococcal cassette chromosome *mec* [15].

Proteins belonging to the PBP family play a crucial role in the formation of the bacterial cell wall and are precursors of the resistance caused by β-lactamase production (see Section “β-Lactamases”).

**Fig. 5:**
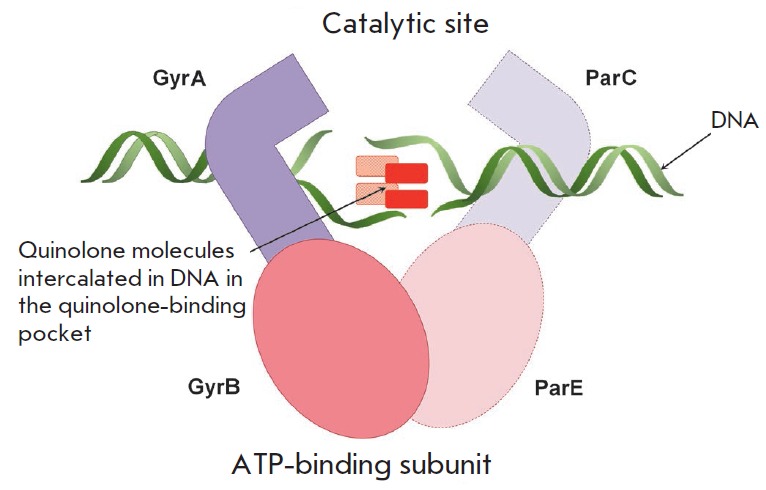
The schematic structure of a ternary complex between type II topoisomerases, DNA, and quinolones. (Gyr A, Gyr B – gyrase subunits, Par C, Par E – topoisomerase IV subunits)

**Type II topoisomerases: DNA gyrase and topoisomerase IV**

Type II topoisomerases include DNA gyrase and topoisomerase IV, which catalyze changes in the spatial configuration of the DNA molecule during replication, transcription, and cell division [16, 17]. DNA gyrase and topoisomerase IV are heterotetrameric enzymes: DNA gyrase consists of two GyrA subunits (97 kDa) and two GyrB subunits (90 kDa); topoisomerase IV consists of two ParC subunits (84 kDa) and two ParE subunits (70 kDa). The GyrA and ParC subunits form the catalytic domains involved in the formation of complexes with the DNA molecule for its break/ligation; the GyrB and ParE subunits exhibit ATPase activity to supply energy to the process.

**Fig. 6:**
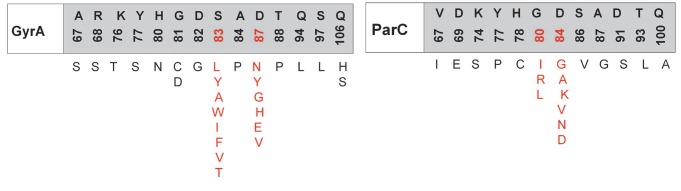
Amino acid mutations in the QRDR region of the GyrA and ParC subunits of type II topoisomerases from *E. coli*, which are responsible for the resistance to quinolones. The color indicates the positions of the mutations whose combination causes a synergistic effect

DNA gyrase and topoisomerase IV serve as targets for quinolones and their derivatives, fluoroquinolones. Formation of the DNA–type II topoisomerase complex is a necessary condition for inhibition (*Fig. 5*). The site of antibiotic binding to the enzyme in the ternary complex is known as the quinolone-binding pocket [17, 18].

The antibiotic binds non-covalently to the active site of the enzyme, so the motion of the enzyme and the replication fork along the DNA molecule is stopped [19]. The formation of the tertiary quinolone–topoisomerase type II–DNA complex stops not only replication, but also transcription, since the motion of RNA polymerase along the DNA template is inhibited [20]. Therein, breaks are formed in the double-stranded DNA molecule, which also determines the bactericidal action of quinolones [21]. Quinolones do not affect mammalian type II topoisomerases, because they differ significantly from bacterial topoisomerases.

The development of quinolone resistance is mainly associated with a reduction in the efficiency of their interaction with the DNA-type II topoisomerase complex due to mutations in the genes, leading to amino acid substitutions in the quinolone-binding pocket. The region of the genes where mutations occur is called QRDR (the quinolone resistance-determining region). These mutations mainly localize to the N-terminal part of the GyrA subunit (the region between residues 67– 106 according to the *E. coli *numbering system) and/ or ParC subunit (amino acid residues 63–102) (*Fig. 6*) but can also affect the GyrB and ParE subunits [18]. The degree of reduction in sensitivity to an antibiotic depends on the mutation type and develops gradually. First, mutations occur in one enzyme and, only later, in another one. A single amino acid substitution at position 67 of the GyrA subunit in *E. coli* increases the MIC of all fluoroquinolones fourfold; at position 81 of the same subunit, eightfold; at position 87, 16-fold; and at position 83, 32-fold [22]. The genes of both subunits carry several mutations, and a synergistic effect is often observed in microorganism strains with a high level of quinolone resistance. Thus, a combination of mutations at GyrA positions 83 and 87 and at ParC position 80 increases the MIC of fluoroquinolones over 4,000-fold [22].

**DNA-dependent RNA polymerase**

The bactericidal effect of rifamycins (rifampin, rifabutin) consists in inhibiting DNA-dependent RNA polymerase [23]. This enzyme consists of five subunits: two α- (molecular weight of each subunit is 35 kDa), β- (155 kDa), β’- (165 kDa), and σ-subunits (70 kDa). The four subunits ββ’αα form the so-called apoenzyme, which exhibits catalytic activity and performs all the main stages of transcription. Transcription initiation and recognition of bacterial gene promoters require the formation of a holoenzyme, which occurs when the regulatory σ-subunit binds to the apoenzyme [24].

**Fig. 7:**
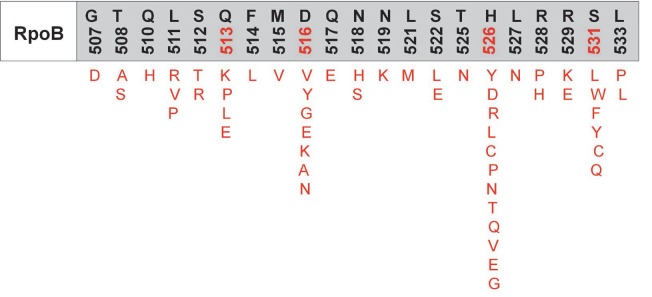
Amino acid mutations in the RpoB fragment of the β-subunit of RNA polymerase, which are responsible for the resistance to rifamycins

Rifamycins selectively bind to the β-subunit of the enzyme near the main channel and inhibit elongation of the originating RNA strand. The emergence of resistance to rifamycins in most cases is associated with mutations in a relatively small fragment of the *rpoB* gene (codons 507–533) encoding the β-subunit of RNA polymerase. Mutations in amino acid residues at positions 513, 516, 526, and 531 (*Fig. 7*) are characterized by the highest degree of polymorphism [25].

**Enzymes catalyzing the biosynthesis of mycolic acids**

The term “mycolic acids” is a generic name for a group of long-chain branched fatty acids, components of the mycobacterial cell wall. Some antituberculosis drugs, derivatives of isonicotinic acid (isoniazid, ethionamide and prothionamide), suppress the synthesis of mycolic acids [25, 26]. These drugs are targeted at enoyl-acyl carrier protein reductase (known as InhA), which is a component of FAS-II fatty acid synthase. It catalyzes the reduction of D_2_-unsaturated fatty acids to saturated ones using the NADPH cofactor as a hydrogen donor [27]. Disrupted synthesis of mycolic acids suppresses the synthesis of the mycobacterial cell wall.

Resistance to these drugs is caused by mutations in the *inhA *gene, which affect either both the promoter region of the *mabA–inhA *operon and cause hyperproduction of the enzyme, or the sequence encoding the enzyme, thus reducing its affinity for the complex between the isonicotinic acid radical and NAD^+^ [28, 29].

## BACTERIAL ENZYMES MODIFYING THE CELL TARGETS OF AMDs

**rRNA methyltransferases**

**Fig. 8:**
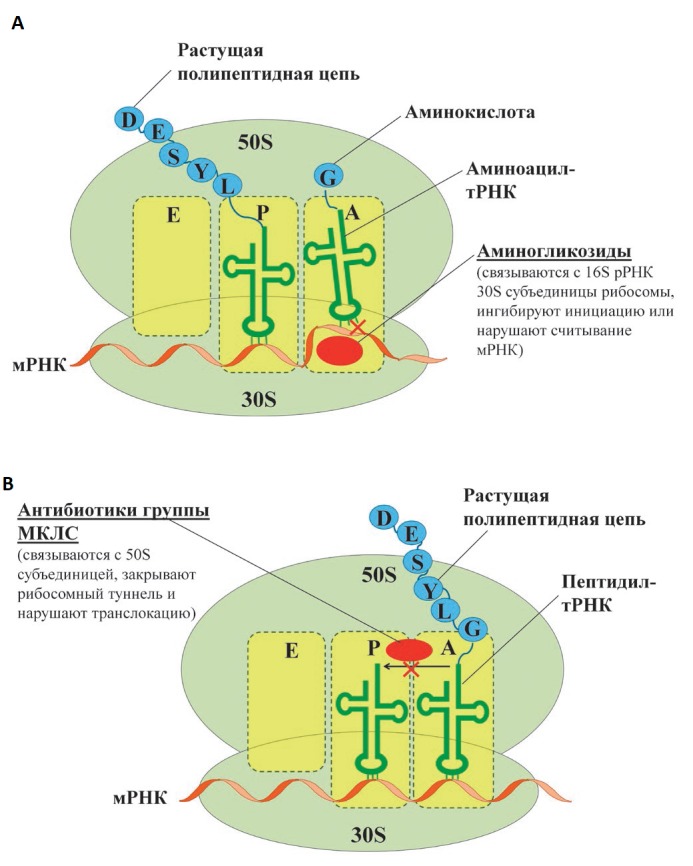
Binding of aminoglycosides (A) and antibiotics of the MKLS group (B) to the ribosome and their effect on protein synthesis

Bacterial ribosomes act as targets for many AMDs [30]. The small 30S subunit consists of 16S rRNA and 21 proteins. Aminoglycosides bind to the 30S subunit to yield hydrogen bonds with the nitrogenous bases of several nucleotides of 16S rRNA, which prevents proper binding of aminoacyl-tRNA to the anticodon and leads to protein synthesis errors and subsequent cell death (*Fig. 8A*). Some aminoglycosides can directly inhibit the initiation or block the elongation of the polypeptide chain [30, 31].

One of the mechanisms of resistance to aminoglycosides is methylation of the A-site of 16S rRNA by bacterial 16S rRNA methyltransferases that results in a loss of the ability to bind to the ribosome by antibiotics [32, 33]. S-adenosyl-*L*-methionine (SAM) donates the methyl group for these enzymes. Eleven different 16S rRNA methyltransferases, which can be divided into two groups according to the type of modified nucleotide in the A-site, have been described. Enzymes classified into the first group (ArmA, RmtA, RmtB, RmtC, RmtD1, RmtD2, RmtE, RmtF, RmtG and RmtH) catalyze the methylation of 16S rRNA at position N7 of nucleotide G1405 and render bacteria resistant only to 4,6-disubstituted aminoglycosides. The second group includes NmpA methyltransferase, which methylates nucleotide A1408 at the N1 position and confers resistance to all known aminoglycosides, except for streptomycin and spectinomycin [31, 32].

The genes encoding these enzymes mainly localize to conjugative plasmids and/or are associated with transposons; they are often linked to other antibiotic resistance genes [34]. The RmtB and ArmA enzymes are the most common. RmtB producers have been isolated not only from clinical specimens of human pathogens, but also from domestic animals, which indicates that resistance determinants can probably be transmitted from animals to humans [33].

Macrolides, ketolides, lincosamides, and streptogramin B (MKLS group according to the name of its components) are targeted at the large 50S subunit of the ribosome containing 5S and 23S rRNA and 33 ribosomal proteins. Despite the differences in their structure, these antibiotics have a common binding site with the 50S subunit in close proximity to the peptidyl transferase center. Meanwhile, they close the ribosomal tunnel, the structural element located in the large ribosomal subunit. This interaction results in dissociation of peptidyl-tRNA from the ribosome, which leads to translocation disruption and termination of protein synthesis (*Fig. 8B*).

One of the mechanisms of resistance to MKLS drugs is the production of 23S rRNA methyltransferases, which catalyze the post-transcriptional modification of 23S rRNA that consists in methylation of A2058 located in the site of antibiotic binding to the ribosome [35]. Like in 16S rRNA methyltransferase, SAM is the donor of the methyl group. Transfer of the methyl group from SAM to A2058 consists of two stages, including the intermediate methylation of the conserved cysteine residue in the C-terminal domain of methyltransferase [36]. Thirty-nine genes encoding 23S rRNA methyltransferase have been described, mainly in Gram-positive microorganisms. In *Enterobacteriaceae*, both chromosomal genes (e.g., *rlmAI*) and the ones localized on mobile genetic elements and encoding ErmB, ErmC, ErmD, ErmE, ErmF, and Erm42 methylases are known. Expression of Erm methyltransferases can be constitutive and inducible. In the constitutive type of expression, synthesis of methyltransferase occurs continuously and does not depend on external conditions. Phenotypically, it manifests itself in resistance to macrolides, lincosamides, and streptogramins B, while ketolides remain active. In the inducible type, methyltransferase is synthesized only in the presence of MKLS. In the absence of an inducer, the regulatory leader sequence of mRNA methyltransferase located in front of the coding sequence has a hairpin conformation and prohibits synthesis of the enzyme. The interaction between the inducer and the mRNA regulatory sequence leads to its rearrangement, which causes the synthesis of methyltransferase.

An active search for efficient inhibitors of rRNA methyltransferase is currently under way. Inhibitors of the SAM-binding center of enzymes mimicking the molecule – donor of the methyl group have been proposed as inhibitors of rRNA methyltransferase but turned out to be non-selective [37]. Compounds inhibiting both the SAM-binding and substrate-binding centers of the enzymes were also proposed [38].

**Enzymes involved in the modification of peptidoglycan in the bacterial cell wall**

Resistance of Gram-positive bacteria to glycopeptide antibiotics (vancomycin and teicoplanin) is caused by the production of enzymes (dihydrogenase, serine racemase, ligase) catalyzing peptidoglycan modification [11]. These antibiotics are high-molecular-weight compounds consisting of glycosylated cyclic or polycyclic peptides. They form a complex with *D*-Ala–*D*-Ala peptidoglycan terminal dipeptide, which is stable thanks to the formation of five hydrogen bonds. Furthermore, these antibiotics prevent the transglycosylation and transpeptidation reactions in the cell membrane (*Fig. 3*) [39]. Resistance to them is caused by substitution of the last amino acid residue *D*-Ala of peptidoglycan for *D*-Lac or *D*-Ser, which reduces the affinity of the terminal dipeptide for the antibiotic (by three orders of magnitude for *D*-Ala-*D*-Lac and by two orders of magnitude for *D*-Ala-*D*-Ser) [40]. Nine operons responsible for the resistance of enterococci to glycopeptide antibiotics have been detected [41, 42]. The *vanA*, *vanB*, *vanD, *and *vanM *operons ensure synthesis of peptidoglycan precursors with the *D*-Ala-*D*-Lac C-terminal dipeptide; the *vanC, vanE, vanG, vanL, *and *vanN *operons ensure synthesis of peptidoglycan precursors with the *D*-Ala-*D*-Ser C-terminal dipeptide [42]. Expression of the products of the aforementioned operons is inducible [43]. The determinants of resistance to glycopeptide antibiotics often localize in plasmids but can also be found in the chromosome.

**Phosphoethanolamine transferases**

**Fig. 9:**
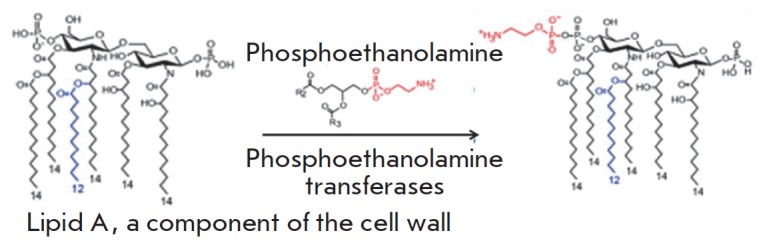
Scheme of modification of lipid A, a component of lipopolysaccharides of the outer cell membrane, by phosphoethanolamine transferase

Polymyxins (colistin) are targeted at the lipopolysaccharides of the outer membrane of Gram-negative bacteria. The main constituent of these AMDs is the positively charged cyclic polypeptide, whose mechanism of action is similar to that of cationic detergents. Interaction between polymyxin molecules and the negatively charged phosphate groups of lipopolysaccharides neutralizes the membrane charge and changes membrane permeability for the intra- and extracellular components. The main mechanism of resistance to polymyxins is associated with closure of the channel of antibiotic penetration into the cell. This channel is closed via the modification of lipid A (the component of lipopolysaccharides) with phosphoethanolamine, which is catalyzed by phosphoethanol amine transferase (*Fig. 9*) [44]. The gene encoding this enzyme has chromosomal localization. The *mcr-1 *gene has recently been detected on plasmids [45]. The development of this type of resistance is associated with mutations in phosphoethanolamine transferase genes [46].

## BACTERIAL ENZYMES MODIFYING AMDS

**Fig. 10:**
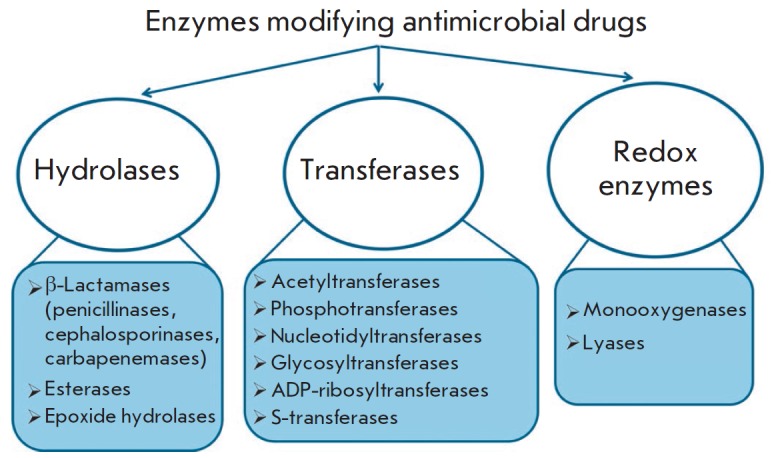
The main classes of enzymes modifying antimicrobial drugs

**Fig. 11:**
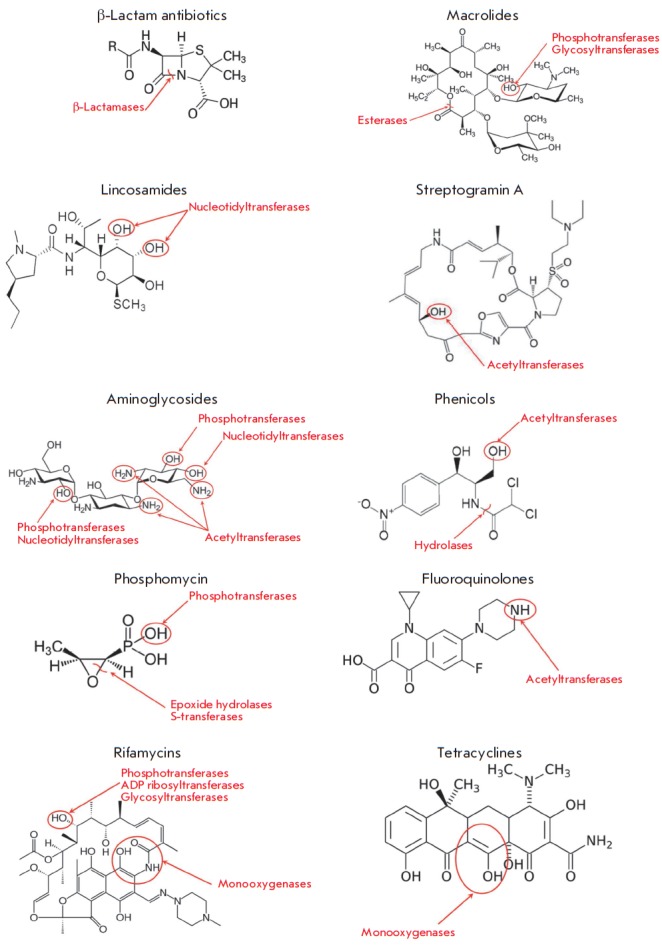
Structures of the main classes of antimicrobial drugs and the enzymes modifying them

Destruction or modification of the antibiotic structure is one of the most common mechanisms of resistance involving enzymes. Depending on the type of reactions they catalyze, the enzymes involved in this resistance mechanism are subdivided into hydrolases, transferases, and oxidoreductases (*Fig. 10*). The structures of the main AMD classes and positions of their enzymatic modification are shown in *Fig. 11*.

**Hydrolases**

β-Lactamase and macrolide esterases destroying β-lactams and macrolides, respectively, are the most common enzymes catalyzing antibiotic hydrolysis. The same mechanism is responsible for the resistance to phosphomycin and chloramphenicol [5, 47].

**β-Lactamases**

β-Lactamases hydrolyze the amide bond in the β-lactam ring, the common structural element of all β-lactam antibiotics (penicillins, cephalosporins, carbapenems, and monobactams). They form an enzyme superfamily that currently consists of more than 2,000 members [47]. According to the homology of amino acid sequences, β-lactamases are subdivided into four molecular classes [48]. The enzymes of classes A, C, and D are serine hydrolases, the enzymes of class B are metalloenzymes.

Serine β-lactamases have structural elements similar to those of the C-domain of PBPs, which indicates that they are evolutionarily related [49]. The evolution of β-lactamase develops via two main mechanisms: the emergence of new mutations in the genes of known enzymes and the emergence of enzymes with a new structure. The high mutation rate of β-lactamases and the localization of their genes on mobile genetic elements contribute to the rapid spread of resistant bacteria, which poses a global threat [50]. Bacteria simultaneously carrying up to eight β-lactamase genes have been detected [51].

Class A β-lactamases (CTX-M, TEM, SHV, and KPC lactamases) are the most common ones [51]. Mutational variability is a feature of TEM and SHV β-lactamases. The key mutations in the active site increase the enzyme volume and make it capable of hydrolyzing the bulk molecules of cephalosporins of the second-to-fourth generations [52]. These mutant forms are known as extended-spectrum β-lactamases (ESBLs). Certain mutations in amino acid residues located at a distance from the active site are compensating and may have multidirectional effects on stability [53, 54].

Class C β-Lactamases efficiently hydrolyze cephalosporins. Initially, this class was represented by the enzymes encoded by chromosomal genes and having an inducible type of expression. Then, enzymes encoded by the genes located on mobile elements were discovered [55].

Class D β-lactamases include OXA-type β-lactamases and are the most structurally diverse enzymes among serine β-lactamases.

The molecular class B is a heterogeneous family of metallo-β-lactamases (MBL) [56]. They contain one or two zinc ions in their active site, hydrolyze almost all β-lactam antibiotics except for monobactams, and are inhibited by chelating agents (EDTA, dipicolinic acid and *o*-phenanthroline). The emergence of new MBL variants (e.g., NDM-type carbapenemases) and their co-expression with serine β-lactamases result in the emergence of bacteria resistant to all β-lactam antibiotics [57].

In order to overcome the resistance caused by production of β-lactamases, an active search for inhibitors of these enzymes is currently under way [58, 59]. In clinical practice, combinations of β-lactams with clavulanic acid, sulbactam, and tazobactam (which contain a β-lactam ring, form a more stable acyl-enzyme complex and have a low deacylation rate) are intensively used to inhibit class A enzymes. The newest inhibitors that are structurally similar to β-lactams but contain no β-lactam ring include diazabicyclooctanes (avibactam and MK-7655). They form carbamyl–enzyme complexes with involvement of catalytic serine, which are then subjected to slow reversible recyclization, accompanied by the release of an inhibitor molecule. These inhibitors have proved effective against A, C, and partly D class β-lactamases. Boronic acid derivatives capable of inhibiting class A carbapenemases are being extensively studied. Particular attention is paid to the search for inhibitors of MBL, but none of them has been used in practice yet [60].

**Macrolide esterases**

Resistance to 14- and 15-membered macrolides (erythromycin, azithromycin, etc.) is caused by the production of esterases catalyzing hydrolysis of the lactone ring [35, 61]. Macrolides containing 16-membered rings are not substrates of these enzymes. Erythromycin esterases EreA and EreB are of the greatest clinical significance. EreA has a more limited substrate specificity profile. It does not hydrolyze azithromycin and telithromycin. It is a metal-dependent enzyme whose activity is inhibited by chelating agents. EreB confers resistance to almost all 14- and 15-membered macrolides, except for telithromycin. The genes encoding these esterases localize in plasmids and are often linked to other antibiotic resistance genes [62].

**Transferases**

Transferases modifying AMD molecules by covalently binding various chemical groups represent a large superfamily of enzymes [5, 6, 63]. Their main groups, differing in terms of substrate specificity, type of modification and mechanism of action, are discussed below.

**Aminoglycoside-modifying enzymes**

Enzymatic modification of aminoglycoside antibiotics is the most common resistance mechanism that is implemented by aminoglycoside-modifying enzymes (AMEs). Several hundred different AME are known; almost each enzyme is represented by several isoenzymes that possess unique substrate specificity and modify aminoglycosides at certain positions [31]. AME genes localize in mobile genetic elements; that is why they rapidly spread.

**Fig. 12:**
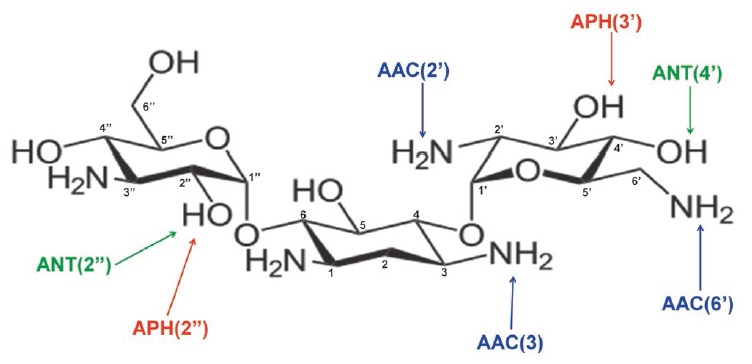
The structure of kanamycin B and positions of its modification by aminoglycoside-modifying enzymes

Three AME families are distinguished according to the reaction type: N-acetyltransferases (AAC), O-phosphotransferases (ANT), and O-adenylyltransferases (ANT) (*Fig. 12*). AAC enzymes use acetyl-CoA as a cofactor; ATP or GTP acts as a donor of phosphate groups and adenine for APH and ANT [23]. AAC enzymes are the most common and clinically significant enzymes; 48 AAC variants acetylating aminoglycosides at one of the positions (1, 3, 2 ’or 6’) have been isolated. The unique Eis enzyme, which is able to simultaneously acetylate aminoglycosides at several positions, is also known.

APH is the second largest family of AME that includes seven types of enzymes catalyzing phosphate group transfer at positions 4, 6, 9, 3’, 2’’, 3’’ or 7” of aminoglycosides. ANT enzymes are divided into five classes modifying aminoglycosides at position 6, 9, 4’, 2’’ or 3’’ [64, 65].

Several approaches have been proposed in order to overcome resistance to aminoglycosides: regulating gene expression by antisense oligonucleotides [66], designing novel aminoglycosides [67, 68], and searching for AME inhibitors [64, 69]. Bisubstrates consisting of aminoglycoside and acetyl-CoA were the first to be proposed as inhibitors of AAC. However, this compound poorly penetrates through the cell membrane and exhibits low effectiveness in *in vivo *experiments because of its considerable size and negative charge [70]. A number of recent studies have shown that AAC and Eis activities are inhibited by the cations of different metals, which increases the effectiveness of aminoglycosides [71]. Various inhibitors of APH possessing kinase activity have been investigated [72]. The natural inhibitor quercetin was found to be among the most effective: it suppresses the activity of several APHs both *in vitro *and *in vivo*. Inhibitors targeted at various AMEs are considered promising: for example, compounds based on 3-(dimethylamino)propylamine inhibit both ANT and APH with sufficient effectiveness [73]. Cationic peptides were bound to the negatively charged active site of AME and exhibited high affinity for different AAC and APH but did not affect resistant bacterial strains, probably due to poor permeability across their cell membrane [74]. The neomycin A dimer inhibited the activity of both monofunctional AAC(6´)- Ii and APH(3´)-IIIa enzymes and the bifunctional AAC(6’)-APH(2”) enzyme, including *in vivo *inhibition using the clinical *Pseudomonas aeruginosa *strain [69, 75].

**Enzymes modifying chloramphenicol and its analogues**

Production of chloramphenicol acetyltransferases (CATs) is the main mechanism of bacterial resistance to chloramphenicol. These enzymes catalyze the addition of the acetyl group of acetyl-CoA to the 3-hydroxyl group of chloramphenicol or its synthetic analogues (thiamphenicol, azidamphenicol), thereby preventing the binding of the antibiotic molecule to ribosomes [5]. CATs do not inactivate fluorophenicol, since the 3-hydroxyl group in its molecule is replaced with a fluorine atom [63]. CATs of different types have extremely low homology of amino acid sequences, which does not exceed 10%. The *cat *genes can be located on chromosomes [76] but are more typically localized on plasmids as components of transposons in association with genes encoding resistance to other AMDs. Expression of the *cat *genes is induced by chloramphenicol [63].

In addition to acetylation, inactivation of chloramphenicol can be ensured by O-phosphorylation. This mechanism of antibiotic resistance was described for *S. venezuelae*, a chloramphenicol producer [77].

**Enzymes modifying MKLS antibiotics**

Macrolide phosphotransferases (MPHs) are enzymes that modify the structure of macrolides by adding a phosphate group to the 2’-OH group [5]. The phosphate group is donated by nucleoside triphosphates, most typically by GTP. Seven different enzymes of this group have been described so far. MPHA preferably catalyzes the phosphorylation of 14- and 15-membered macrolides, while MPHB modifies 14- and 16-membered macrolides [35, 62]. The genes encoding MPH are located on mobile genetic elements containing other genes encoding resistance to macrolides and other antibiotic classes [78, 79]. Expression of the genes coding for macrolide phosphotransferases can be either inducible (*mphA*) or constitutive (*mphB*) [35].

Macrolide glycosyltransferases are enzymes that inactivate macrolides by glycosylating the 2’-OH group of the macrolide ring [6]. They use UDP glucose as a cofactor.

Streptogramin acetyltransferases inactivate only streptogramins A by acetylation of an unbound hydroxyl group; their mechanism of action is similar to that of CAT [5]. The genes encoding these enzymes have been identified in a number of Gram-positive pathogens, including staphylococci and enterococci [63].

**Phosphomycin-modifying enzymes**

FosA, FosB, and FosX epoxidases, as well as FomA and FomB kinases, are metalloenzymes that inactivate phosphomycin [11, 23, 80]. Epoxidases open the epoxy group of phosphomycin (the oxirane ring) by adding various substrates. FosA is glutathione-S-transferase that uses Mn^2+^ and K+ metal ions as cofactors, besides glutathione. Bacillithiol or *L*-Cys acts as a source of the thiol group in FosB; additionally, these enzymes use Mg^2+^ as a cofactor [11, 81]. The FosX enzyme is a Mn^2+^-dependent hydrolase. Most of the genes encoding these enzymes localize on the plasmid, although FosA in *P. aeruginosa *and FosB in *S. aureus *are encoded by chromosomal genes.

FomA and FomB kinases add one or two phosphate groups to the phosphomycin molecule, using ATP and Mg^2+^ ions as cofactors. These enzymes are isolated from phosphomycin producer *S. wedriensis* [11].

**Rifamycin-modifying enzymes**

Several groups of enzymes inactivate rifamycins by modifying the hydroxyl group, the key group involved in the binding of an antibiotic molecule to the β-subunit of RNA polymerase. NAD^+^-dependent enzymes belonging to the Arr group catalyze ADP-ribosylation, RPH kinases catalyze phosphorylation, and glycosyltransferases catalyze glycosylation [23, 82, 83].

**Monooxygenases**

The flavin-dependent monooxygenase TetX confers resistance to all tetracyclines, including the broad-spectrum antibiotic tigecycline [5]. TetX catalyzes monohydroxylation of tetracyclines in the presence of NADPH, O_2_, and Mg^2+^, leading to intramolecular cyclization and decomposition of the molecule. Flavin-dependent monooxygenases Rox inactivate rifamycins by oxidating the naphthyl group at position 2, leading to ring opening and linearization of the antibiotic molecule [84].

**Enzymes of metabolic processes modifying AMD in the prodrug form**

Antibiotics can also be modified by the enzymes that protect cells against toxic molecules. In most cases, prodrug forms of AMDs are modified to the active forms.

Isoniazid is activated by KatG catalase-peroxidase, giving rise to free radicals of isonicotinic acid, which block the enzymes involved in the synthesis of mycolic acids [85]. Resistance is caused by mutations in the *katG *gene, which are most often localized in codon 315 and cause conformational changes in the isoniazid-binding pocket.

Structural analogues of isoniazid, ethionamide and prothionamide, are activated by NADPH-dependent FAD-containing monooxygenase encoded by the *ethA *gene [85]. The oxidized forms of ethionamide and prothionamide in a complex with NAD^+^ inhibit the enzymes responsible for the synthesis of mycolic acids (primarily InhA), similar to the case of isoniazid. Expression of the *ethA *gene is regulated by the transcriptional repressor EthR. Resistance is caused by mutations in the *ethA *and *ethR *genes.

## BIFUNCTIONAL ENZYMES: A NEW EVOLUTIONARY TREND

Mutations in bacterial genomes and selection of new resistant phenotypes are the main mechanisms in bacteria responsible for antibiotic resistance. As a result, there is a wide variety of forms causing resistance for a number of enzymes: thus, over 2,000 β-lactamases have been described. However, single amino acid substitutions cause limited changes in the activity and specificity of a particular enzyme. The emergence of bifunctional enzymes encoded by two linked genes is a new trend in the evolutionary development of resistance. This phenomenon significantly increases substrate specificity and provides evolutionary advantage for extremely broad resistance to various AMDs [86].

**Bifunctional β-lactamases**

The first bifunctional enzyme Tp47 was isolated from the causative agent of syphilis *Treponema palladium *[87]. It has two active sites: one exhibiting PBP activity; and the second one, β-lactamase activity. Since Tr47 has a very low β-lactamase activity, it does not really provide resistance to β-lactams.

Another bifunctional β-lactamase, blaLRA-13, was found in β-lactam-resistant *E. coli *strains isolated from Alaskan soils [88]. This enzyme consists of 609 amino acids, which is almost twice greater than the length of the typical monofunctional β-lactamase. The C-domain of this enzyme (356 amino acids) is highly homologous to class C β-lactamases and ensures resistance to amoxicillin, ampicillin, and carbenicillin, while the N-domain (253 amino acids) is highly homologous to class D β-lactamases and ensures resistance to cephalexin. In addition to blaLRA-13, the isolated strains also produced several monofunctional β-lactamases belonging to different classes. Although this bifunctional β-lactamase has not yet been found in clinical bacterial strains, one cannot rule out the possibility that it will be distributed among infectious human diseases in the future. Moreover, the discovery of this enzyme confirms the evolutionary hypothesis that soil microorganisms, as well as microorganisms of other ecological niches, have a wide range of resistance mechanisms that can be transferred over time to clinically significant pathogens.

Enzyme bifunctionality could have occurred during the evolutionary changes in high-molecular-weight dual-domain PBP, whose transpeptidase domain can form a stable complex with β-lactam antibiotics. During mutation emergence, the binding site became able to hydrolyze the β-lactam ring; i.e., a new group of antibiotic-hydrolyzing enzymes was formed.

**Bifunctional aminoglycoside-modifying enzymes**

Gram-positive bacteria were found to have a bifunctional AAC(6’)-Ie/APH(2”)-Ia enzyme. The N-terminal domain of this enzyme possesses acetyltransferase activity, while the C-domain exhibits phosphotransferase activity [89]. The AAC domain of the enzyme can acylate only one type of aminoglycoside rings, while the APH domain has broader specificity and catalyzes the O-phosphorylation of four different aminoglycoside rings [90]. A bifunctional enzyme ensures resistance to almost all known, clinically significant aminoglycosides, except for streptomycin and spectinomycin.

The bifunctional ANT(3’’)-Ii/AAC(6’)-IId enzyme is characterized by a combination of nucleotidyltransferase activity against streptomycin and spectinomycin and acetyltransferase activity with broad substrate specificity [91].

The first domain of the bifunctional AAC(3)-Ib/ AAC(6’)-Ib’ enzyme is specific only to gentamicin and fortimicin; the second domain exhibits broad substrate specificity, including amikacin, dibekacin, gentamicin, isepamicin, kanamycin A, and neomycin [92].

A novel bifunctional enzyme, AAC(6’)-30/AAC(6’)- Ib’, providing resistance to many aminoglycosides other than isepamicin and exhibiting higher activity than monofunctional enzymes, has recently been isolated from *P. aeruginosa *[93].

**Bifunctional aminoglycoside- and fluoroquinolone-modifying enzyme**

**Fig. 13:**
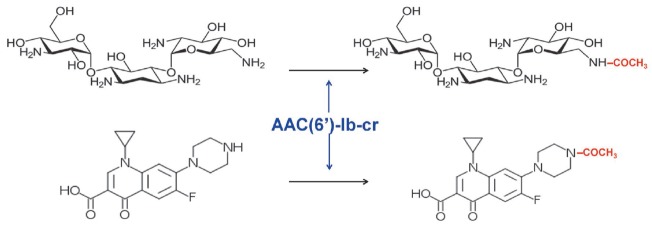
Acylation of kanamycin B and ciprofloxacin catalyzed by the bifunctional enzyme AAC(6’)- Ib-cr

A novel variant of AAC(6’)-Ib-cr acetyltransferase is the first enzyme that can simultaneously inactivate aminoglycosides and fluoroquinolones (*Fig. 13*) [94]. Two mutations encoding the W102R and D179Y substitutions ensure ciprofloxacin resistance [95]. The gene coding for this enzyme has both plasmid and chromosomal localization. It was found on a multi-resistant plasmid, together with other resistance genes.

## CONCLUSIONS

The question regarding the origin of the bacterial enzymes responsible for resistance development during evolution remains controversial. The genes encoding these enzymes are located on chromosomes and mobile elements. The enzymes encoded by chromosomal genes protect microorganisms producing antibiotics against modification of their potential targets. Resistance occurs when the genes coding for these enzymes are transferred to other bacteria.

Another group of enzymes encoded by chromosomal genes has evolved from enzymes belonging to superfamilies with isolation of subgroups with altered substrate specificity. Enzymes that perform vital functions and are responsible for the biosynthesis of cell wall polysaccharides, proteins, nucleic acids, and metabolites serve as targets for antibiotics. Modification of the active sites of target enzymes contributed to their ability to use antibiotics as substrates. The presence of proto-resistance genes causing the evolutionary relationship between β-lactamases and PBP, kinases and acetyltransferases, with aminoglycoside-modifying enzymes, has been established.

Many enzymes have originated from bacterial pro-enzymes that used to have other functions. Mutations in the genes encoding enzymes emerged due to exogenous and endogenous factors (in particular, antibiotics and products of their metabolism). These mutations changed the structure, catalytic properties, and substrate specificity of these products. The multiplicity of mutations indicates that both the key and accompanying amino acid residues undergo mutations. The key amino acid residues are important for catalytic processes, while changes in the accompanying residues compensate for structural changes and function as allosteric sites of activity regulation

The multidirectionality of the processes is a feature typical of bacterial resistance. Combination of several resistance mechanisms in a single cell (e.g., modification of structural cellular elements, changes in the expression level of proteins, including porins, and activation of efflux systems) complicates the development of methods for suppressing resistance. The scientific concept of combining objects related to the most important biological processes into certain groups has emerged in recent years. Thus, the concept of “microbiome” as a combination of microorganisms of a certain species and humans appeared. Non-pathogenic microorganisms, and soil bacteria in particular, represent a huge reservoir and source of resistance genes. Their wide distribution among microorganisms is associated with localization on plasmids and other mobile genetic elements and a high rate of exchange and transmission between bacterial cells, including pathogenic strains.

The combination of the genes responsible for the resistance of pathogenic clinical strains and non-pathogenic bacteria in the environment and microbiota is known as the “resistome.” Its important feature is that the genome of a single bacterium carries several resistance genes that ensure multiresistance. Bacterial cells can rapidly reproduce, change their gene structure, and undergo selection; so, they have developed new mechanisms ensuring cell survival. Enzymes with various functions play the most important role in these processes. The term “enzystome” can be used to refer to the enzyme-based defense system that has developed throughout the long-term evolution of bacteria.

The presented classification of bacterial enzymes of the “enzystome” will be further developed and supplemented. Having summarized the results of analyzing the contribution of enzymes to the development of antibiotic resistance in bacteria, one should acknowledge the fundamental biological significance of this process as it ensures the survivability of microorganisms and their adaptability. The adaptability of microorganisms to new environmental conditions largely depends on “biocatalytic functionality.” We believe that microbiologists, molecular biologists, and biotechnologists should focus closely on changes in this functionality at the genetic level. The growing industrial-scale production of AMDs and their uncontrolled use in medicine and veterinary medicine has become a powerful anthropogenic factor which has significantly contributed to the acceleration of resistance development. Research into the structures of the enzymes that compose the “enzystome” and the analysis of evolutionary variability and the conservative sites of the “resistome” will allow us to understand the mechanisms of regulation in bacterial cells and to find new targets for developing rational approaches to the production of selective and effective AMDs in order to overcome resistance. It is of particular interest to use enzymes capable of destroying and metabolizing antibiotics as medications to protect the beneficial microbiota and prevent side effects during antibiotics therapy.

## Abbreviations

AMD: antimicrobial drugs
AME: aminoglycoside modifying enzymes
ESBL: extended spectrum ?-lactamases
MBL: metallo-?-lactamases
MKLS: macrolides, ketolides, lincosamides and streptogramins
PBP: penicillin-binding proteins
AAC: aminoglycoside-N-acetyltransferases
ANT: aminoglycoside-O-adenylyltransferases
APH: aminoglycoside-O-phosphotransferases
CAT: chloramphenicol acetyltransferase
MPH: macrolide phosphotransferases
NAG: N-acetylglucosamine
NAM: N-acetylmuramic acid
QRDR: quinolone resistance determining region
SAM: S-adenosyl-L-methionine
